# A Bayesian Driver Agent Model for Autonomous Vehicles System Based on Knowledge-Aware and Real-Time Data

**DOI:** 10.3390/s21020331

**Published:** 2021-01-06

**Authors:** Jichang Ma, Hui Xie, Kang Song, Hao Liu

**Affiliations:** State Key Laboratory of Engines, Tianjin University, Tianjin 300072, China; majichang@tju.edu.cn (J.M.); songkangtju@tju.edu.cn (K.S.); spartaaa@163.com (H.L.)

**Keywords:** convolutional neural network, sensing environment, cognitive understanding, dynamic Bayesian networks, human driver agent, decision-making, autonomous vehicle, lane changing behavior

## Abstract

A key research area in autonomous driving is how to model the driver’s decision-making behavior, due to the fact it is significant for a self-driving vehicles considering their traffic safety and efficiency. However, the uncertain characteristics of vehicle and pedestrian trajectories affect urban roads, which poses severe challenges to the cognitive understanding and decision-making of autonomous vehicle systems in terms of accuracy and robustness. To overcome the abovementioned problems, this paper proposes a Bayesian driver agent (BDA) model which is a vision-based autonomous vehicle system with learning and inference methods inspired by human driver’s cognitive psychology. Different from the end-to-end learning method and traditional rule-based methods, our approach breaks the driving system up into a scene recognition module and a decision inference module. The perception module, which is based on a multi-task learning neural network (CNN), takes a driver’s-view image as its input and predicts the traffic scene’s feature values. The decision module based on dynamic Bayesian network (DBN) then makes an inferred decision using the traffic scene’s feature values. To explore the validity of the Bayesian driver agent model, we performed experiments on a driving simulation platform. The BDA model can extract the scene feature values effectively and predict the probability distribution of the human driver’s decision-making process accurately based on inference. We take the lane changing scenario as an example to verify the model, the intraclass correlation coefficient (ICC) correlation between the BDA model and human driver’s decision process reached 0.984. This work suggests a research in scene perception and autonomous decision-making that may apply to autonomous vehicle system.

## 1. Introduction

Intelligent cognitive understanding and anthropomorphic decision-making are core technical problems that must be solved to realize autonomous driving. The human driver is a complex intelligent agent that has the ability to think, summarize its experience and continuously optimize and improve its driving behavior. The decision-making process of a human driving is a dynamic response to the surrounding traffic scene, which can be divided into three processes: scene cognition; inference decisions; and automatic execution. In recent years, several studies have been conducted on the driving agent, that can be classified into three primary categories: traditional rule-based formula methods; learning-based end-to-end methods and probabilistic reasoning methods.

Although rule-based algorithms such as the if–else rules encompass the current state-of-the-art approaches in autonomous driving, they cannot fully cope with the complexity and uncertainty of traffic elements in the urban road environment.

The second learning-based approach relies on convolutional neural networks (CNN) and GPU-related computation [1,2] In the context of a driver agent model for an autonomous vehicle system, a typical approach of the end-to-end model is based on a deep neural network with a supervised learning algorithm, which is trained to predict the human driver’s control command (steer angle, etc.) when encountering the same observation in traffic scene images. Successful applications of this method include the ALVINN system in [3], the DAVE system described in [4], and the Dave-II system [5,6]. Although deep neural networks (DNNs) provide an efficient way to form an autopilot system, it is still difficult to deal with complicated traffic scenarios and adapt with different driving maneuvers. At the same time, the end-to-end agent model usually depends on a large-scale driving video dataset or data augmentation process in order to improve the generalization ability of the model [7]. Otherwise, the agent will learn a poor performance.

Instead of the end-to-end learning-based agent model, researchers have begun to focus on inference decision autonomous vehicle systems. A dynamic Bayesian network (DBN) approach was used to realize the simulation of the driver’s inference decision process based on knowledge-aware and real-time data. References [8,9,10] proposed a driving decision awareness model which can infer driving behaviors such as lane changing. The data required to train the agent model are generated by human drivers. It is capable of dealing with special situations and generates the expected planning and control strategy. The results demonstrated that the Bayesian network can transfer human skills to the intelligent assistance system [11]. Modeling of driving behavior based on inference methods makes the decision model interpretable, which overcomes the challenge of the black box characteristics of the end-to-end networks. As a result, the theoretical approach combined with end-to-end on the basis of supervised learning and the inference intention is necessary to model the driver decision behaviour and we have designed our vision-based autonomous vehicle system with learning and inference method within this framework. Based on the above literature analysis, we desire a mathematical representation that can directly simulate driving decision behavior, which can deal with complex traffic scene cognition and where the decision-making process is interpretable, rather than blindly mapping the traffic image to steering angles. In order to solve these problems, this paper propose a Bayesian driver agent (BDA) model for autonomous vehicle systems based on knowledge-aware and real-time data, which is inspired by the human drivers’ cognitive psychology. The focus of this research is to model drivers’ decision behavior through the effective integration of a convolutional neural network (CNN)’s predictive ability and a dynamic Bayesian network (DBN)’s causal reasoning mechanism, forming an intelligent agent for autonomous vehicle systems. The perception module which is based on a multi-task learning neural network (CNN) takes a driver’s-view image as its input and predicts the traffic scene feature values. The decision module which is based on a dynamic Bayesian network (DBN) then makes an inference decisions using traffic scene feature values. The model can learn the lane-change behavior of drivers and produce the optimal driving mode by calculating the expected confidence. In general, the BDA model should:

- Sense and cognitively understand the current traffic scene situation;
- Predict the confidence and probability distribution of current driving patterns;
- Process partially observable and uncertain information.

To demonstrate the reliability and validity of the BDA model, we performed hardware-in-loop experiments on a driving simulation platform. The BDA model effectively realized scene cognitive understanding and decision reasoning. Compared with the decision-making process of human drivers, the intraclass correlation coefficient reached 0.984. It also provides a sound technical support for the autonomous decision-making of intelligent driving vehicles.

The rest of the paper is organized as follows: the detailed approach of the BDA model is described in Section 2; experiments and simulation results are given in Section 3; the discussion is presented in Section 4; the conclusions and future research work are presented in Section 5.

## 2. Approach for the Bayesian Driver Agent Model

As the human pilot drives the vehicle, two functional regions of their brain—cognitive understanding and inference decisions—are activated. First, the cognition region receives information in the form of traffic images and achieves the goal of understanding the current driving situation by extracting the scene indicator values. Second, the inference region receives the indicator information and executes real-time reasoning, obtaining the maximum posterior probability of a decision under the current situation. This description leads us to answer a key research objective: to numerically simulate the autonomous decision-making process of drivers to achieve a human-like driving strategy.

**Figure 1:**
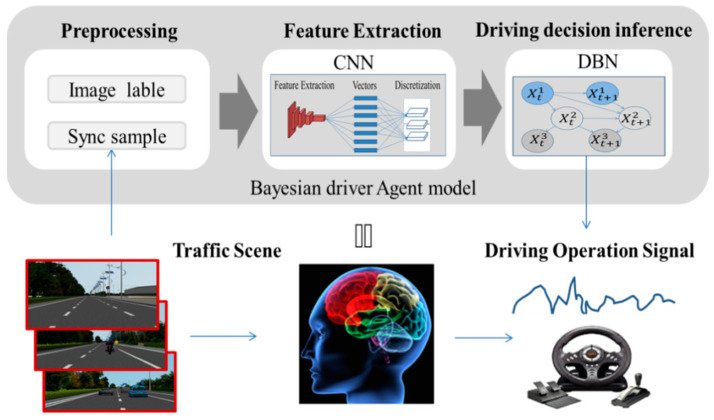
Principle of the driving decision model diagram.

In order to meet this objective, we used a multi-layer CNN to process the traffic scenario images, simulate the cognitive function region and predict the indicator values of the road scenes. The probabilistic model DBN was employed to simulate the reasoning function region, receive the predict indicator values and calculate the confidence of the decision under the current situation. The proposed BDA decision architecture consists of two cooperating submodules: the scene feature extraction network and probabilistic causal reasoning network. An algorithmic logic diagram of the BDA model is shown in Figure 1.

Scene cognition understanding can be described as a mapping function from the traffic scene image to the scenario situation factor [12]. To implement this function, we defined the CNN training task as multi-label learning used for predicting the indicator values of traffic situations, such as estimations of the indicator values of the longitudinal distance between the ego car and the traffic car (e.g., front, front left, and front right). The data set used for CNN training was obtained from our driving simulator platform.

From a neurological point of view, the neocortex makes decisions in the belief space, so it is more reasonable to use a probabilistic model of human intelligence agents, as mentioned in the literature [13,14]. Probabilistic graph networks provide a manner of reasoning under uncertain conditions and can effectively integrate the driver’s a priori knowledge. In addition, the network node operation is based on a causal reasoning algorithm so that the decision-making process can have interpretable characteristics. We selected standard variables, such as the vehicle speed, longitudinal acceleration and heading angle, to construct a vector space for the driving decision. 

### 2.1. Conventional Neural Network-Based Simulation of a Human Driver Agent’s Cognitive Functional Region

**Figure 2:**
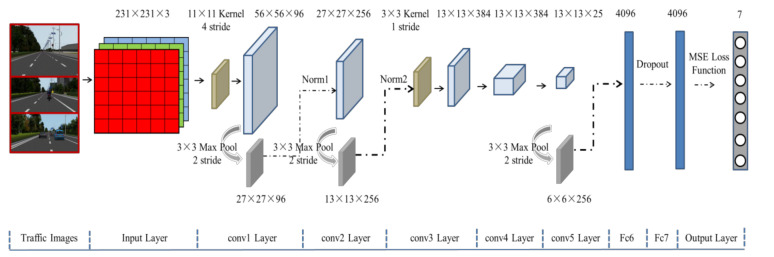
Schematic of the convolutional neural network (CNN).

The main task for the perception module is to extract useful features from the traffic scene images and achieve the purpose of understanding the current driving situation. The previous CNN works perceive the underlying features from the driver’s first-view scene image, which include longitudinal distance between the ego-vehicle and other traffic vehicles, distance to lane boundary markings [12], lane boundary marking detection and lane position estimation [15]. In fact, these features show strong visual correlation. For example, longitudinal distance is used to judge the condition of lane changes to avoid collisions with obstacles. The distance from the center of the rear axle of the vehicle to the lane boundaries, and distance to the line markers on the left and right sides of the current lane are feature information that can be used to calculate the current position of the vehicle. In this paper, we focus on urban road driving with three lanes. In order to utilize these scene features and improve learning performance, we define the perceptual problem as a multi-label learning task in the convolutional neural network (CNN) framework, which has been successfully applied in many fields, such as [16] which proposed a method to jointly model object detection and distance prediction based multi-task combination strategy. The effectiveness of the implementation of the agent’s decision depends on the accuracy of its understanding of the environment. The CNN-enabled method is effective for modeling cognition in complex environments. Therefore, an image was mapped to several meaningful description values of the scene, rather than being directly mapped to steering wheel angles like the end-to-end network [5,6]. We adopted a state-of-the-art deep neural network with nine convolutional layers (as shown in Figure 2) to train the network and predict feature values of a traffic scene.

The CNN was based on the Caffe deep learning framework and the standard CNN architecture [1,2]. It contains eight layers, including five convolutional layers and three fully-connected layers, which involves convolution (conv), max pooling (pool), normalization (norm) operations and dropout strategies. To make the entire network structure clearer, we will briefly introduce these contents.

Assume that the input of the convolutional neural network CNN is the original scene image *P*, $F_{i}$ represents the feature map of the *i*-th layer and the calculation process of $F_{i}$ can be described as:

(1) $$F_{i}=f\left(F_{i-1}\;⊗\;W_{i}+b_{i}\right)\;\;\;\;\left(1\leq i\leq 5\right),$$

in the formula, $\;W_{i}$ represents the weight vector of the *i*-th layer,$\;b_{i}$ is the offset vector of the *i*-th layer, ⊗ means that the convolution kernel is used to convolve the feature map of the *i*−1-th layer, and finally the feature map $F_{i}$ of the *i*-th layer is obtained through the nonlinear activation function *f.* The network uses ReLU (rectified linear unit) as the nonlinear activation function of neurons, and the mathematical expression of the ReLU function is:

(2) $$f\left(x\right)=max\left(\begin{array}{cc} 0, & x \end{array}\right),$$

The pooling layer follows the convolutional layer to down-sample the feature map and prevent overfitting, the pooling operation can be described as:

(3) $$F_{i}=sub_{down\_sample}\left(F_{i-1}\right),$$

in the formula, $sub_{down\_sample}$ is a down-sample function described in the literature [17,18], and the maximum pooling function is used to perform feature sampling after the conv1, conv2, and conv5 convolutional layers.

Norm represents the normalization of local response, the response-normalized activity $b_{x,y}^{i}$ is given by the expression:

(4) $$b_{x,y}^{i}=a_{x,y}^{i}/{\left(k+\alpha \sum_{j=\max\left(0,i-n/2\right)}^{\min\left(N-1,i+n/2\right)}{\left(a_{x,y}^{i}\right)}^{2}\right)}^{\beta },$$

where $a_{x,y}^{i}$ is a neuron computed by applying kernel *i* at position (*x*, *y*) and then applying the ReLU non-linearity, *n* is the size of the normalization neighborhood and *N* is the total number of kernels in the layer. The constants *k, n, α*, and *β* are hyper-parameters whose values were pre-set: *k* = 2, *n* = 5, $\alpha \;=10^{-4}$, and β = 0.75.

The dropout strategy is to set the output of each hidden neuron to zero with probability 0.5, this strategy is used in the last two fully-connected layers of CNN to alleviate the overfitting problem and improve the generalization ability of the learning model [19].

Summarizing the process in Figure 2, the first convolutional layer filters the 231 × 231 × 3 input image by through 96 kernels of size 11 × 11 with a step of 4 pixels, and then we get 56 × 56 × 96 feature maps. The second convolutional layer takes the output of the first convolutional layer (norm and max pool) as input and filters it with 256 kernels of size 5 × 5 with a step of 1 pixels. The third, and fourth convolutional layers are connected to each other without any intervening pooling or normalization layers, the third convolutional layer has 384 kernels of size 3 × 3 connected to the outputs of the second convolutional layer. The fourth convolutional layer has 384 kernels of size 3 × 3, and the fifth convolutional layer has 256 kernels of size 3 × 3. Each fully-connected layer has 4096 neurons. The output value of the last fully connected layer is passed to the output layer, MSE loss function is used to calculate the error between the predicted value and the genuine lable. Processed by five convolutional layers and three fully connected layers, traffic images are mapped to seven indicators. In actual computation, the sky background information of the traffic scene image is redundant and does not contribute to the robustness of the model. Therefore, the input image was resized to 231 × 231. The structural parameters of each layer are shown in Table 1.

The gradient descent algorithm was used for the training, and the updating rules of weight ω were as follows:

(5) $$v_{i+1}≔0.9\cdot v_{i}-0.0005\cdot \varepsilon \cdot \omega_{i}-\varepsilon \cdot \langle \frac{\partial L}{\partial \omega }|\omega_{i}\rangle D_{i},$$

(6) $$\omega_{i+1}≔\omega_{i}+v_{i+1},$$

where *v* is the momentum, ε is the learning rate, and $\langle \frac{\partial L}{\partial \omega }|\omega_{i}\rangle D_{i}$ is the stochastic gradient decay term. The output of the CNN is the predicted indicator values of the scenario. The schematic and meaning of the indicators are discussed below.

**Figure 3:**
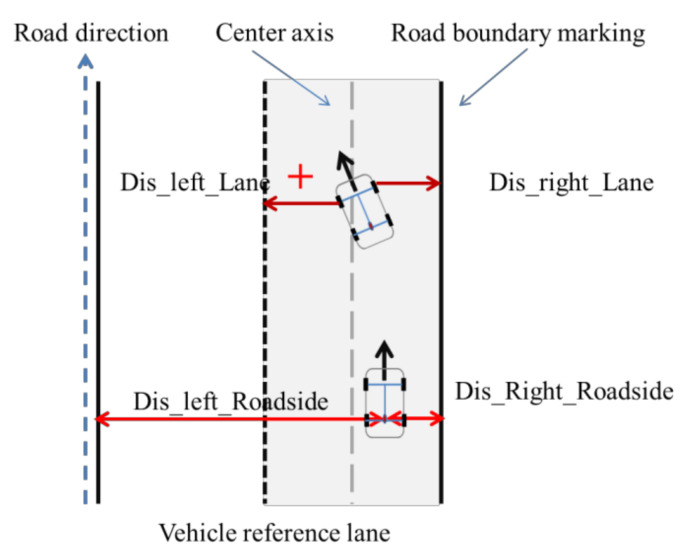
Horizontal safe distance for scenario representation.

**Figure 4:**
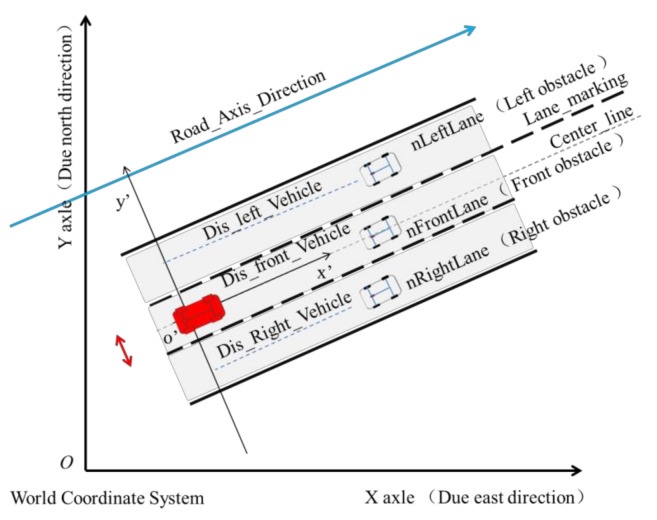
Longitudinal safe distance for scenario representation.

From the driver point of view, we only need to understand the traffic situation in its current lane and the two adjacent (left/right) lanes when making decisions, so we select seven indicators related to decision-making to describe the current driving situation, the specific meaning of each indicator is shown in Figure 3 and Figure 4, and Table 2. To account for traffic regulations, the lane center line should be fit to ensure the safety of vehicles. Therefore, the road boundary and lane mark were selected as the references for the horizontal distance (Figure 3).

Furthermore, the driver must consider the effect of the traffic between the vehicle and the ego-vehicle. The longitudinal safe distance was established via coordinate transformation (Figure 4), where *XOY* is the road coordinate system and $\;x^{\prime }o^{\prime }y^{\prime }$ is the vehicle coordinate system.

To summarize, a total of seven parameters constitute the scenario indicators. The specific meanings of the parameters are shown in Table 2.

During the training phase, we collected the traffic scene images from our driving simulator platform, and recorded the synchronized ground truth indicator values at an interval of 50 ms. Here, we used the mean square loss function to train the network, which is defined as:

(7) $$Loss=\frac{1}{n}\sum_{k=1}^{k=n}{\left(y_{k}-x_{k}\right)}^{2}.$$

The output of CoveNet is vector $x_{k}$, which is composed of seven estimation indicator values. $y_{k}$ represents the ground truth indicator values. The results of the CNN simulation of the human driver agent’s cognitive function region are analyzed in Section 3.1.

### 2.2. Dynamic Bayesian Network-Based Simulation of a Human Driver Agent’s Inference Functional Region

As for the decision-making process, the human pilot responds to the traffic situation around the ego-vehicle according to their own driving experience and habits. In order to explore the relationship between the traffic scenario indicator input and the agent decision output, we employed the DBN to numerically simulate the human pilot decision-making process based on priori knowledge and environmental real-time observation data.

The Bayesian network is a directed acyclic graph in which nodes represent variables and arcs represent the dependencies between nodes [8]. The random variable is $X=\left\{X^{1},X^{2},\cdots ,X^{n}\right\}$, where $X^{i}$ stands for a node in the network structure, $P_{a}\left(X^{i}\right)$ represents the parent node of $X^{i}$, and $X^{i}$ at time *t* is expressed as $X_{t}^{i}$. The joint probability distribution of *X* is:

(8) $$P\left(X^{1},X^{2},\cdots ,X^{n}\right)=\prod_{i=1}^{n}P(X^{i}|P_{a}\left(X^{i}\right)).$$

**Figure 5:**
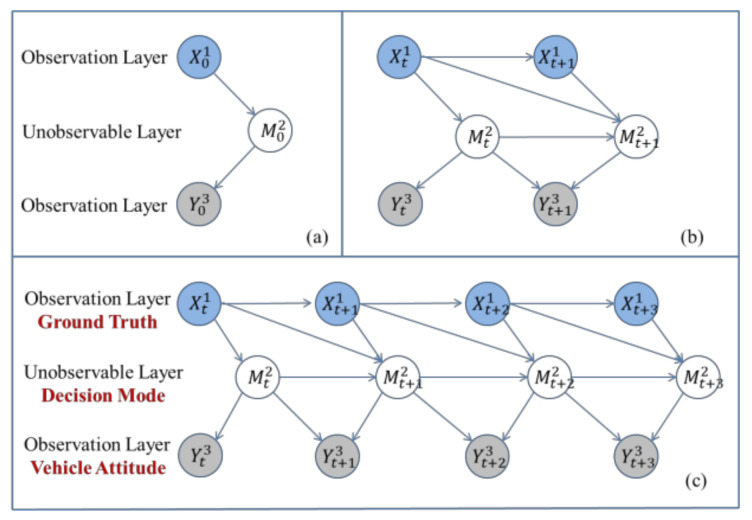
Schematic of DBN: (**a**) DBN initial network B_0; (**b**) DBN transition network B_→; and (**c**) DBN expanded into three time slices.

The structure of the DBN model was obtained by extending the BN model with time. Time stamps are discrete independent variables; we built a local model for each time slice, which is shown as three time slices in Figure 5c [20].

The DBN is composed of two parts: an initial network, $B_{0}$, which is defined as the prior probability distribution on variable $X_{t}^{i}$, and a state transition network, $B_{\to }$, which is defined as the transition probability distribution $P(X_{t+1}^{i}|X_{t}^{i})$ on variable $X_{t}^{i}\to X_{t+1}^{i}$.

Therefore, for a given DBN structure, we computed the joint probability distribution of an arbitrarily node on $\left\{X_{1},X_{2},\cdots ,X_{T}\right\}$ as:

(9) $$P\left(X_{1:T}^{\left(1:N\right)}\right)=\prod_{i=1}^{N}P_{B_{0}}(X_{1}^{i}|P_{a}\left(X_{1}^{i}\right))\;\times \;\prod_{t=2}^{T}\prod_{i=1}^{N}P_{B_{\to }}(X_{t}^{i}|P_{a}\left(X_{t}^{i}\right)).$$

In the DBN application area, the key research problem is finding the best possible structure of a network ($\;S_{DBN}$) that fits the sample dataset $D=\left\{X_{1},X_{2},\ldots ,X_{n}\right\}$, i.e., the maximum value ($P\left(S_{DBN}|D\right)$) of the directed acyclic graph:

(10) $$P\left(S_{DBN}|D\right)=\frac{P\left(S_{DBN}\right)P(D|S_{DBN})}{P\left(D\right)}.$$

The data likelihood of a given network structure can be calculated with relevant network parameter θ:

(11) $$P\left(D|S_{DBN}\right)=\int P(D|S_{DBN},\theta )P(\theta |S_{DBN})d\theta .$$

In the actual calculation process, its approximate value is:

(12) $$logP\left(D|S_{DBN}\right)=log\;P\left(D|S_{DBN},\hat{\theta_{S}}\right)-\frac{1}{2}\log N*\#S,$$

where $\hat{\theta_{S}}$ represents the optimal parameter estimation, which is used for maximizing the data likelihood of $S_{DBN}$; *N* is the instance variable of the sample data set; and #S stands for the number of parameters:

(13) $$\#S=\frac{\pi_{i}\left(\gamma_{i}-1\right)}{2},$$

where $\pi_{i}$ is the state number of the parent node and $\gamma_{i}$ is the state number of the child node. As for DBN, the network parameter is $\#S=\#S_{0}+\#S_{\to }$.

The structure learning of the Bayesian network involves obtaining the logical relation of each variable. Representative research achievements include the K2 algorithm and the Bayesian measurement mechanism [21,22]. In recent years, more intelligent algorithms have been used to realize structure learning, such as the genetic algorithm [23], and the application of reinforcement learning in BN structure learning [24].

The state space grows exponentially as the number of nodes increases. Therefore, it is not possible to prevent the above algorithms from exploring a large space. In order to reduce the high dimensional exploration space, we introduced an expert knowledge constraint-based greedy search algorithm called KB-GES. In the actual computation, the prior conditional probability is used to express the expert knowledge, and subsequently, the bayesian information criterion (BIC) $BIC\left(S:D\right)=BIC_{0}+BIC_{\to }$ scoring function is improved via the following equations:

(14) $$BIC_{0}=\sum_{i}\sum_{j}\sum_{k}N_{i,j,k}^{0}\cdot log{\hat{\theta }}_{i,j,k}^{0}-\frac{1}{2}logN\cdot \#S_{0}+N\cdot \frac{1}{2}log\left(1+\frac{e}{N}\right),$$

(15) $$BIC_{\to }=\sum_{i}\sum_{j}\sum_{k}N_{i,j,k}^{\to }\cdot log{\hat{\theta }}_{i,j,k}^{\to }-\frac{1}{2}log\cdot \#S_{\to },$$

where $N_{i,j,k}$ represents the number of samples satisfying the child node variable $X_{i}=k$ and the parent node variable $\pi \left(X_{i}\right)=j$ in the instance dataset *N*, and $\left(X_{i},\pi \left(X_{i}\right)\right)$ is the local family structure formed by the variable $X_{i}$ and its parent node set $\pi \left(X_{i}\right)$, which represent the contribution of instance data to the likelihood function. The optimal parameter $\hat{\theta }$ is estimated using the standard maximum likelihood as follows:

(16) $${\hat{\theta }}_{S}^{0}={\hat{\theta }}_{i,j,k}^{0}=\frac{N_{i,j,k}^{0}}{\sum_{k}N_{i,j,k}^{0}},$$

(17) $${\hat{\theta }}_{S}^{\to }={\hat{\theta }}_{i,j,k}^{\to }=\frac{N_{i,j,k}^{\to }}{\sum_{k}N_{i,j,k}^{\to }}.$$

The parameter e in Equation (10) is the prior conditional probability (CPT) constraint of experts on the relationship of node variables. The pseudo-code of the KB-GES algorithm flow is shown in Algorithm 1.

**Algorithm 1** KB-GES based on the fusion of priori knowledgeInput: ρ: Variable order; e:  Experts constraints; μ: Maximum number of parent nodes; D: Complete sample data.Output: Optimal Bayesian network structure.1: G ←boundless graph composed of nodes $X_{1},X_{2},\ldots ,X_{n}$
2: for *j* = 1 to *n*3:   $\pi_{j}\leftarrow \;\emptyset \;;\;V_{old}\leftarrow BIC\left(\left(X_{j},\pi_{j}\right)|D\right)$
4:   while (True)5:   $\;i\leftarrow \arg max_{1\leq i\leq j,x_{i}\notin \pi_{j}}BIC\left(\left(X_{j},\pi_{j}\cup \left\{X_{i}\right\}\right)|D\right)$
6:   $\;V_{new}\leftarrow BIC\left(\left(X_{j},\pi_{j}\cup \left\{X_{i}\right\}\right)|D\right)$
7:   $\;if\;(V_{old}\leftarrow V_{new}\;and\;\left|\pi_{j}\right|<\mu )$
8:      $V_{old}\leftarrow V_{new};$
9:      $\pi_{j}\leftarrow \pi_{j}\cup \left\{X_{i}\right\};$
10:      Add an edge$\;X_{j}\leftarrow X_{i}$ to G11:   else 12:    break;13:   end if14:  end while15: end for16: return G

The ground truth and vehicle attitude information is the observable random variable, while the driving decision-making is a pilot-neural activity in the brain, which belongs to unobservable random variables, as shown in Figure 5c. In the actual driving process, the human pilot first receives the ground truth indicator values of the traffic scene, and subsequently generates decisions according to their own subjective experience and driving intention. We selected the standard variable, for instance, the vehicle’s speed, longitudinal acceleration, and course angle, to construct a vector space for driving decisions. The specific meanings of the parameters are shown in Table 3. The driving mode discretization values are shown in Table 4.

The training database consisted of scenario indicator values and a driving decision vector, and then KB-GES algorithm was used to learn the DBN network structure in the sample database. After that, the posterior probability of the decision query node, which is called belief updating, was calculated. Finally, the DBN output the maximum expected posterior confidence of lane keep, lane change (left or right), and drive-free. The results of the DBN simulation of the human driver agent’s inference function region will be analyzed in Section 3.2.

## 3. Experiments and Analysis of Results

**Figure 6:**
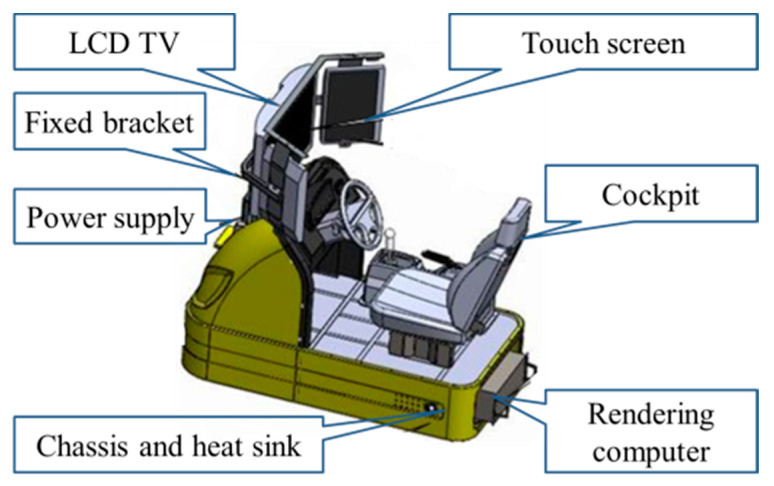
Schematic of the simulation platform structure.

We conducted a hardware-in-the-loop test on a driving simulator to analyze the effectiveness of the proposed Bayesian driver agent model. A schematic diagram of the simulator is shown in Figure 6.

**Figure 7:**
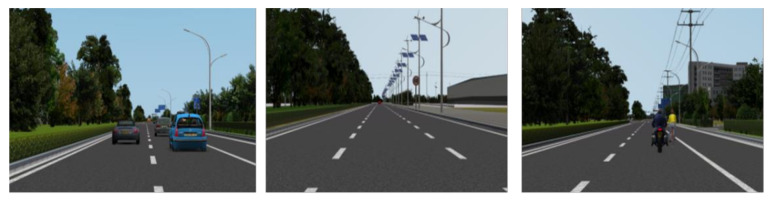
Schematic diagram of traffic scenario simulation.

The platform uses simulation technology to integrate the visual system (LCD TV, touch screen) and the cockpit. On the rendering computer, we have developed the models for five urban roads, based on which a total of 1000 test cases were designed. Examples for the test cases are shown in Figure 7.

**Figure 8:**
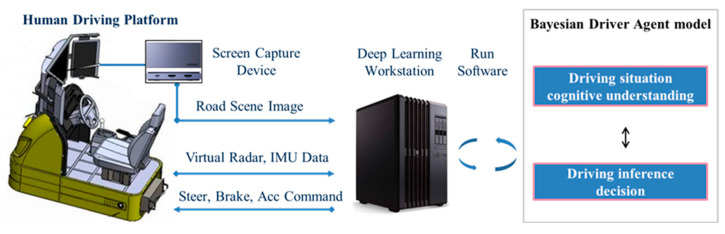
Schematic of the data generation system.

As shown in Figure 8, the simulation platform mainly contains three parts: human driving platform, screen capture device, and Bayesian driver agent software.

The human driving platform provides virtual radar and IMU data, we can parse out obstacle distance and vehicle attitude information, as well as road genuine indicator values. The simulator platform can also executes control commands (steer, brake, acc) from the BDA model through a dedicated API function. The screen capture device is used to record synchronized scene images and serve the images as training data for the convolutional network. The Bayesian driver agent model runs on deep learning workstation, which composed of two submodules. First of all, the cognition module achieves the goal of understanding the current driving situation by extracting the scene indicator values. Second, the inference module receives the indicator information and executes real-time decision inference, the final calculation result is returned to the driving simulator. 

**Figure 9:**
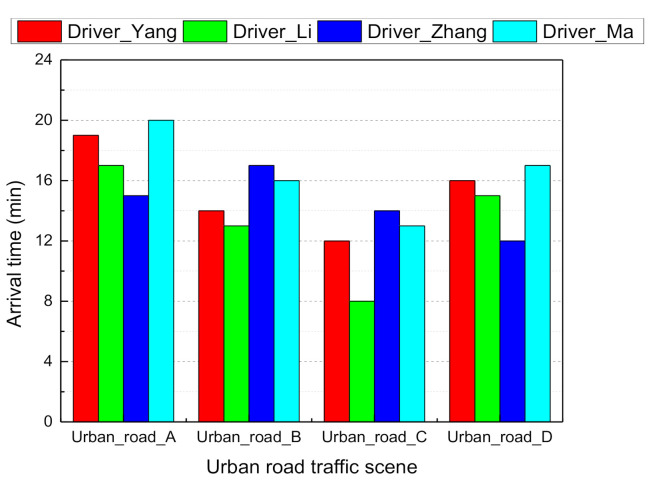
Histogram of the task completion time for each urban road.

**Figure 10:**
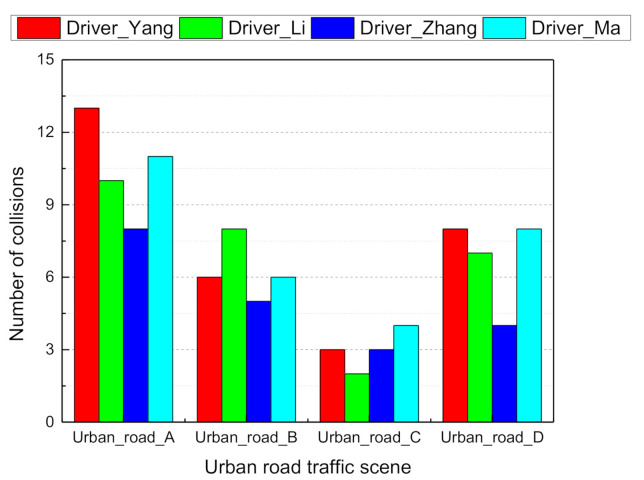
Number of collisions during driving for each urban road.

Four volunteers were selected to drive the simulator manually in order to collect traffic scene images and synchronous environment ground truth indicator values. set the acquisition frequency to 50 ms. We performed a qualitative evaluation of the driving task completion time (Figure 9) and the number of collisions (Figure 10) during the driving simulation in the urban road traffic scene. Drivers who took less than 15 min to complete the entire road segment and less than five collisions were labeled as “good drivers”, and their data were saved as a positive sample database (e.g., driver Zhang). A total of 69,000 data samples were available for learning the driver agent model. At each time step, the CNN model took a driving scene image from the simulator screen and estimated the affordance indicators and the DBN model then processed the indicators and computed the joint probability distribution of the driving mode.

### 3.1. Cognitive Ability with Multi-Layer Convolutional Networks

**Figure 11:**
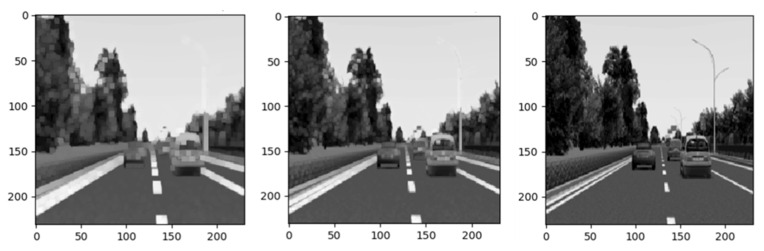
Application of morphological filtering in image pre-processing.

In the training phase, to build our training set, we manually drive a virtual vehicle on the simulator to collect screenshots (driver’s first perspective) and the corresponding ground truth values of the selected seven feature indicators. This data were stored and used to train a CNN in a supervised learning manner. In the testing phase, at each time step, the trained network takes a driving scene image from the simulator and estimates the indicator values to achieve cognitive understanding of the current traffic situation. We use a state-of-the-art deep learning CNN as our direct perception model to map an image to the feature indicators. In actual computation, the sky background information of the traffic scene image is redundant and does not contribute to the robustness of the model. Therefore, the input image was resized to 231 × 231. And then morphology filter [25] is used to pre-process the scene image to enhance the quality of scene images and improve the feature information of regions of interest (ROI), the pre-processing result is shown in Figure 11.

**Figure 12:**
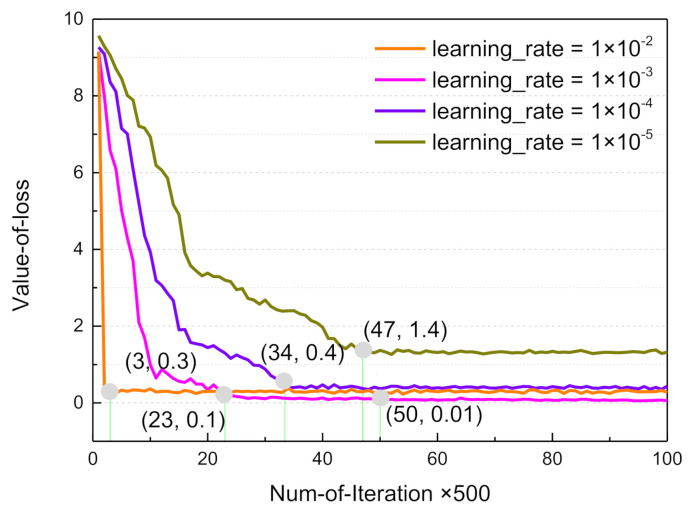
Network loss values for different learning rates.

Our direct perception CNN was based on the Caffe deep learning framework and the standard CNN architecture to automatically learn image features for estimating feature indicators related to driving decision. It contains eight layers, including five convolutional layers and three fully-connected layers. MSE loss is used as the loss function. The direct perception CNN architecture provides an approach for scene understanding in autonomous driving. The scene description ground truth data were used to train the CNN model, in order to realize a cognitive understanding of the traffic situation. As described in Equations (1) and (2), the learning rate ε is directly related to the convergence speed and prediction accuracy of the network. Therefore, during the training phase, we fine-tuned the learning rate in the range of [1 × 10^−2^, 1 × 10^−3^, 1 × 10^−4^, 1 × 10^−5^]. From Figure 12, it can be seen that the effective learning rate was 1 × 10^−3^, rapid network convergence was achieved after 11,500 iterations, and the value of the loss function decreased to 0.1. Finally, the network converged to the target value of 0.01 after 25,000 iterations.

**Figure 13:**
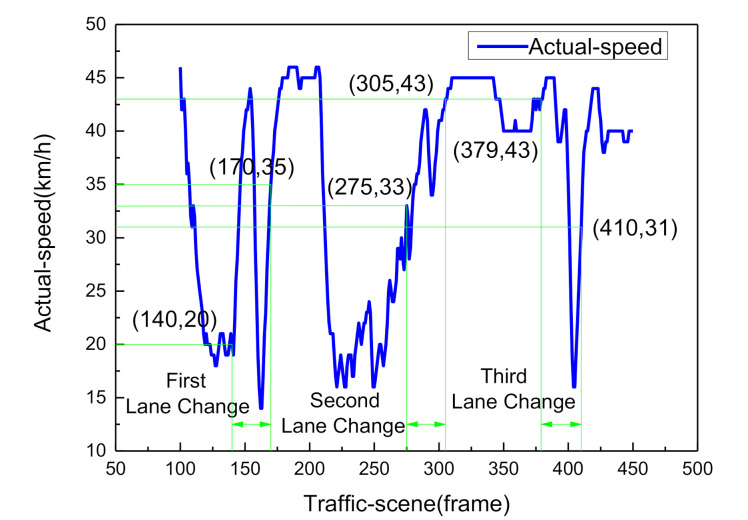
Actual speed curve of the lane-change scene.

In order to measure the accuracy of estimation for indicators, we constructed a lane-change testing case, as shown in Figure 13.

**Figure 14:**
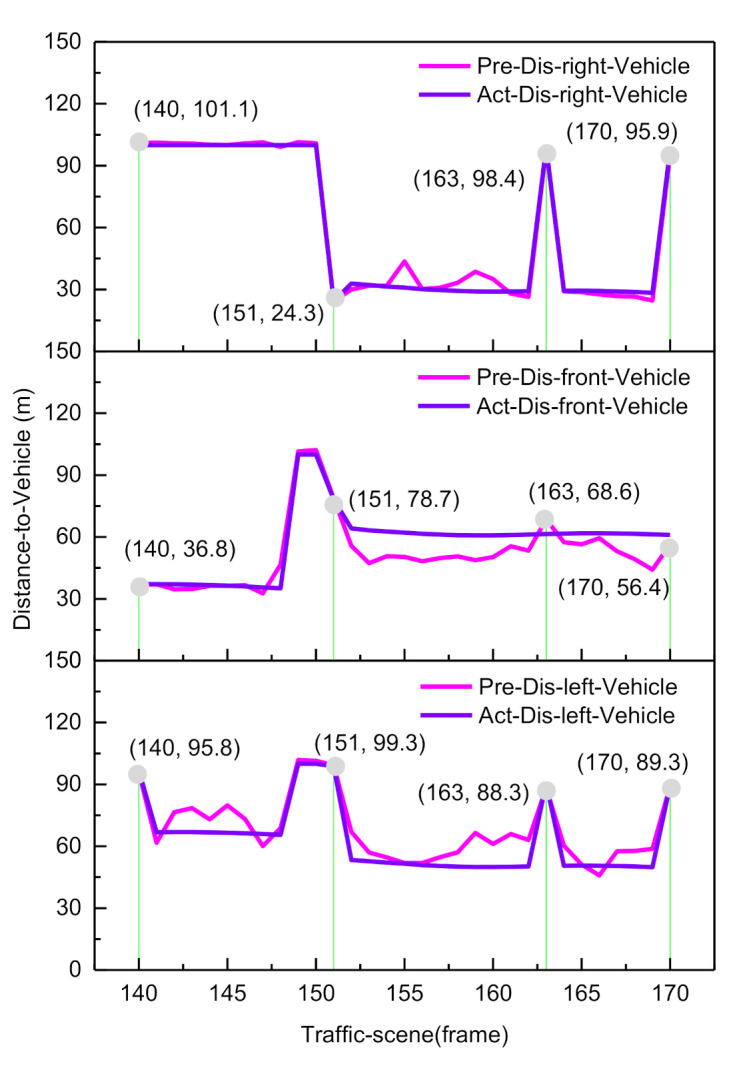
Estimation of the longitudinal scene description factor.

**Figure 15:**
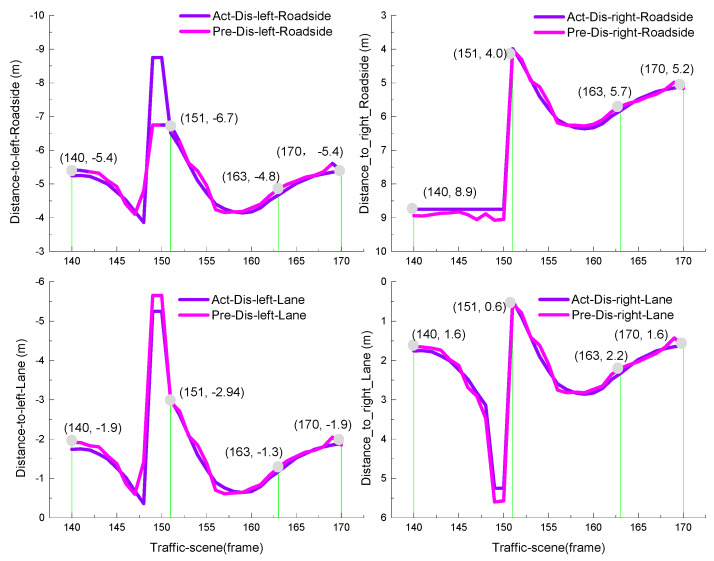
Estimation of the horizontal scene description factor.

In Figure 13, the first set of lane-change occurred from frame 140 to frame 170, the second set of lane-change occurred from frame 275 to frame 305, and the third set of lane-change occurred from frame 379 to frame 410. In this study, we took the first lane-change data segment for analysis. The comparison of the actual ground truth indicator value (blue line) and the CNN estimated indicator value (pink line) is illustrated in Figure 14 and Figure 15.

During the lane-change behavior process, the longitudinal distance information of a vehicle in traffic directly affects the human pilot’s driving decision when overtaking. At frame 140, the distance of the obstacle in the current lane is 36.8 m, and the distance of the obstacle in the left lane is 95.8 m, so the human driver’s left lane-change intention is generated.

**Figure 16:**
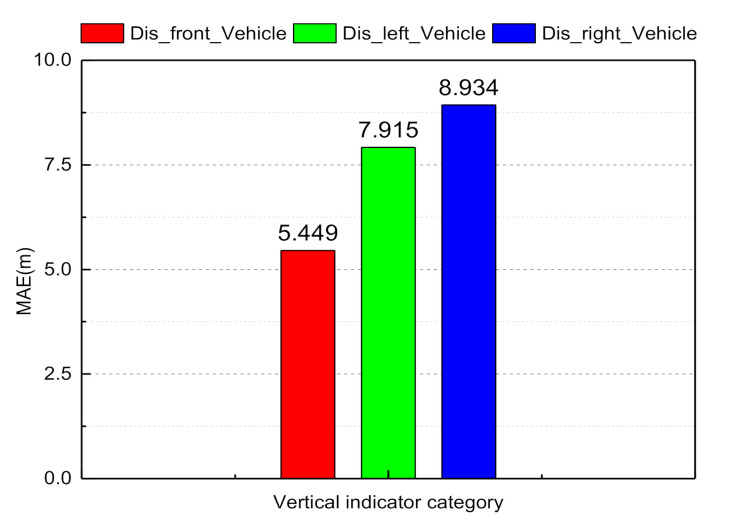
Mean absolute error histogram of the longitudinal scene indicator values.

**Figure 17:**
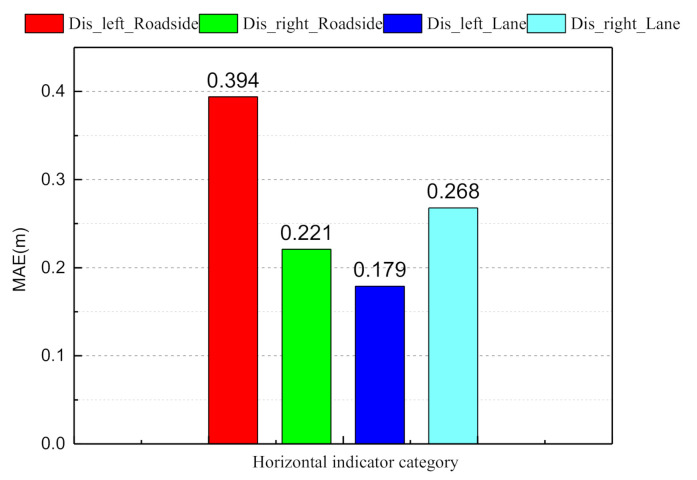
Mean absolute error histogram of the horizontal scene indicator values.

The horizontal distance information determines whether the vehicle stays on the road and keeps the center line running. From frame 140 to frame 163, the ego-vehicle position changes from the center line of the current lane to the target lane. At frame 170, the vehicle completes the left lane change and maintains the center line, which can be seen from Figure 15. The distance from the vehicle to both sides of the lane marker is 1.9 and 1.6 m, and the distance from the vehicle to both sides of the road boundary is 5.4 and 5.2 m. We used the mean absolute error (MAE) between the ground truth values and the estimated values to evaluate the CNN prediction ability:

(18) $$MAE=\sum_{i=1}^{N}\left|y_{i}^{estimate\_value}-y_{i}^{ground\_truth}\right|,$$

where N=7 is the size of the indicators. From Figure 16 and Figure 17, it can be observed that the longitudinal indicator MAE is less than 9 m and the horizontal index MAE is less than 0.5 m. In summary, the BDA agent model can accurately predict the indicator values of the complex urban road traffic scene. This result implies that the model has a cognitive understanding of driving situations.

**Figure 18:**
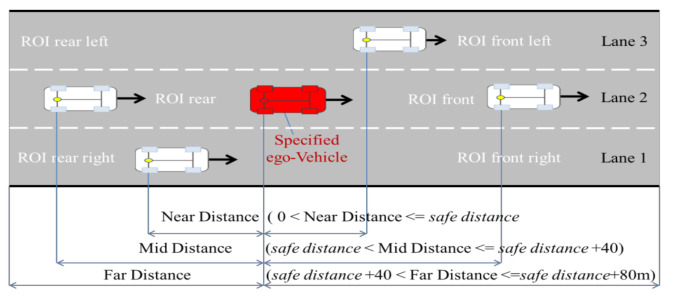
Diagram of the longitudinal distance node state for a three-lane road, where the ego-vehicle is currently in lane 2.

The output indicator values of the convolutional network are regarded as the Bayesian network node variable. Since the units and ranges of each variable are different, we added a discretization layer (Table 1). The continuous observations were discretized by the fuzzy method. Several discretization methods have been compared in the literature [26,27,28]. The results show that the discretization process improved the prediction performance of the BN model. In other words, it made the uncertain reasoning more interpretable. In this paper, we used the S membership function to discretize the continuous indicator values into {near_distance, mid_distance, far_distance}, which is defined as:

(19) $$f\left(x_{i},a,b,c\right)=\left\{\begin{array}{c} \begin{array}{cc} 0, & {\;x}_{i}\leq a\\ 2{\left[\left(x_{i}-a\right)/\left(c-a\right)\right]}^{2} & \;a<x_{i}\leq b \end{array}\\ \begin{array}{cc} 1-2{\left[\left(x_{i}-a\right)/\left(c-a\right)\right]}^{2} & b<x_{i}\leq c\\ 1,\; & {\;x}_{i}>c \end{array} \end{array}\right\},$$

where a is the safe lane-change distance and its calculation result is a function of the vehicle’s speed, as shown in the following formula:

(20) $$S=\frac{{\left(\frac{{\mathrm{veh}}_{—}\mathrm{Speed}}{3.6}\right)}^{2}}{2*g*\mu }\;+\;{\mathrm{veh}}_{—}{\mathrm{Speed}*t}_{\mathrm{driver}},$$

where g = $9.8m/s^{2}$ and $t_{\mathrm{driver}}$ is the reaction time of the driver and its value is in the range of 0.5–0.6. µ is taken as 0.8. c is the forward pre-sighting distance, and b = (a + c)/2. The value range of each state is shown in Figure 18.

After obtaining the CNN output indicator values, the membership degree of the fuzzy set was obtained by inserting it into the membership degree function, which is more consistent with the mindset of human beings to make decisions based on fuzzy values rather than specific math distance values. Similarly, the horizontal distance and vehicle’s attitude were also discretized by the S function.

### 3.2. Inference Decision with Dynamic Bayesian Networks

The basic task of inference involves calculating the maximum posterior probability of driving decision nodes based on real-time indicator values of a traffic scene, which is called belief updating. As explained in Section 2.2, in this experimental work, we first set up an a priori network structure for off-line qualitative analysis based on a priori-knowledge, and then implemented the proposed structure learning algorithm KB-GES to learn the network structure based on real-time data for on-line quantitative analysis.

(a) A priori network structure based on expert experience

The a priori network structure is defined according to the observable variables, the including ground truth value and vehicle attitude information, resulting in a total of 20 node variables. The specific meaning of each node variable is illustrated in Figure 3, Figure 4, and Figure 18. The representative nodes of DBN are described in Table 5.

**Figure 19:**
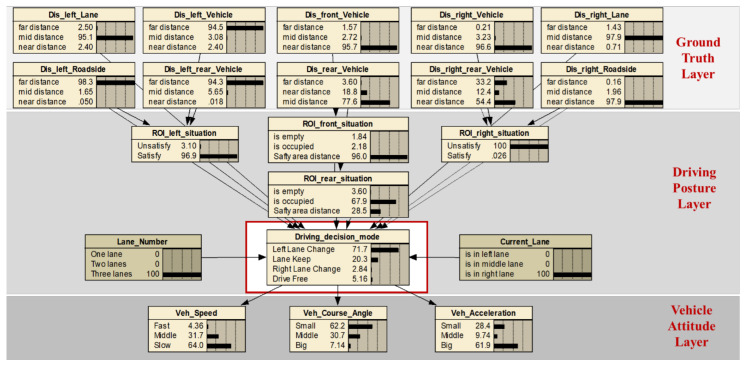
Architecture diagram of a priori Bayesian network.

Based on the first intuition of driving experience, the node corresponding to the driving decision mode is associated with all observable variables and is a qualitative analysis of the driver’s decision-making process (Figure 19). The initial conditional probability table (CPT) is set a priori to fit the decision-making thought process of human drivers.

Note that the proposed prior structure is an extension of naive Bayes, which is only used to compare the structures obtained by the automatic structure learning algorithm. The ground truth layer nodes combined with the driving posture layer nodes are input to the driving decision mode node, and this process belongs to the positive probability propagation to update the confidence of the driving decision mode. Vehicle attitude layer nodes are child nodes of the driving decision mode node, so inverse probability propagation is applied to update the confidence of the driving decision mode.

**Figure 20:**
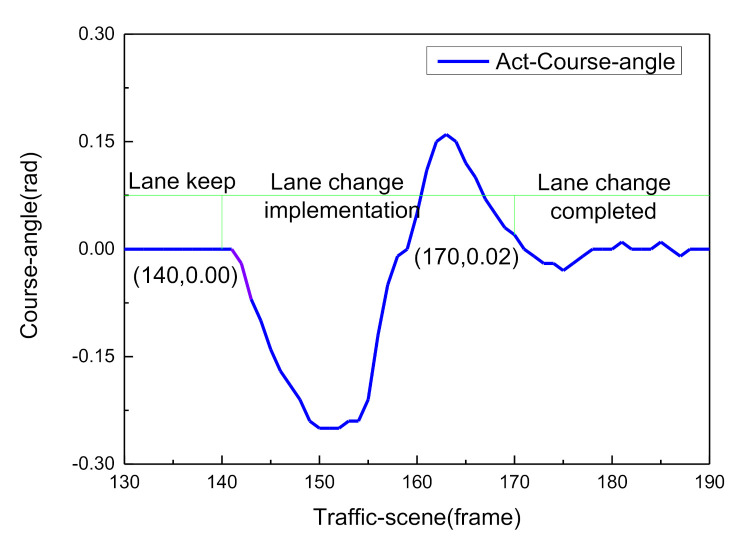
Change of the vehicle course angle during lane change.

**Figure 21:**
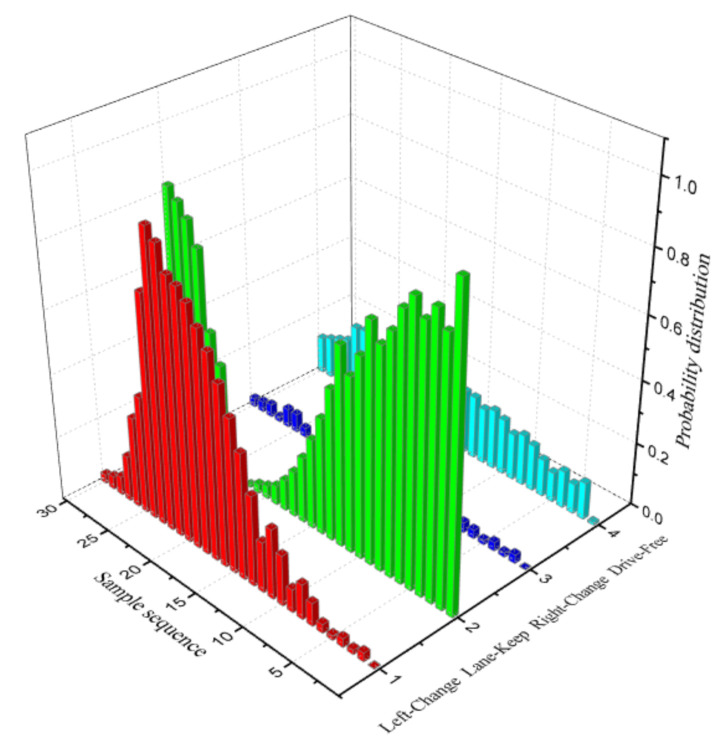
Probability distribution of the driving decision mode.

This paper takes the first left lane-change case for analysis. A total of 31 sampling points were taken from the 140th frame to the 170th frame, which is typically divided into three stages of lane changing motivation generation, lane changing implementation, and lane changing completion, as shown in Figure 20. The posterior probability distribution of the driving decision mode is shown in Figure 21.

From 1–10 sampling points, the probability distribution of Lane_Keep remained within 0.54–0.75. Therefore, the lane maintenance mode was performed first. When the front vehicle entered the safe area, the distance between the ego-vehicle and the front traffic vehicle reached 36.8 m. Meanwhile, when the obstacle in the left lane was at a distance of 95.8 m (Figure 13), the driving situation satisfied the left lane changing condition, so from 11 to 23 sampling points, the probability distribution of Left_Lane_Change gradually increased from 0.17 to 0.71. As a result, the left lane-change decision mode was executed. Subsequently, by adjusting the attitude of the vehicle to enter the lane-keeping mode again, from sampling points 24 to 30, the probability distribution of Lane_Keep gradually increased from 0.21 to 0.71, and the full lane-change decision was then executed. The results show that the posterior probability distribution confidence of the driving decision mode is consistent with the experimental setting, conforming to the three stages of driver lane change (Figure 20). The qualitative analysis demonstrates the effectiveness of the DBN reasoning model.

(b) Structure learning from sample data using the KB-GES algorithm

**Figure 22:**
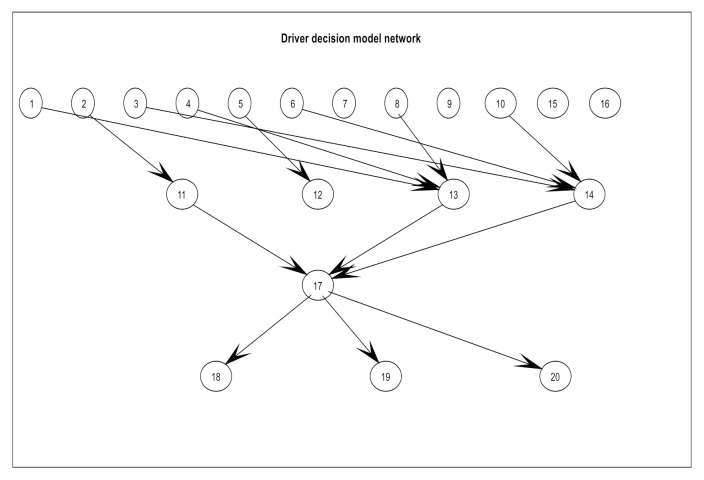
DBN structure learned from sample data.

The purpose of structural learning is to obtain the relationship between each variable that affects the driving decision, which is known to be an NP-hard computing problem [29]. To this end, we used our proposed KB-GES algorithm to learn the Bayesian network structure from driving data, which has been discussed in Section 2.2. The software and hardware used for this purpose were Ubuntu 16.04 and an Nvidia1080 GPU. Respectively, for the programming and implementation of the BDA model, we used ProBT a C++ Library API and Murphy’s BNT toolkit for co-programming, which is free for academic use [30,31]. The driver graph structure learned based on sample data is shown in Figure 22, the meaning of each node is shown in Table 5. 

**Figure 23:**
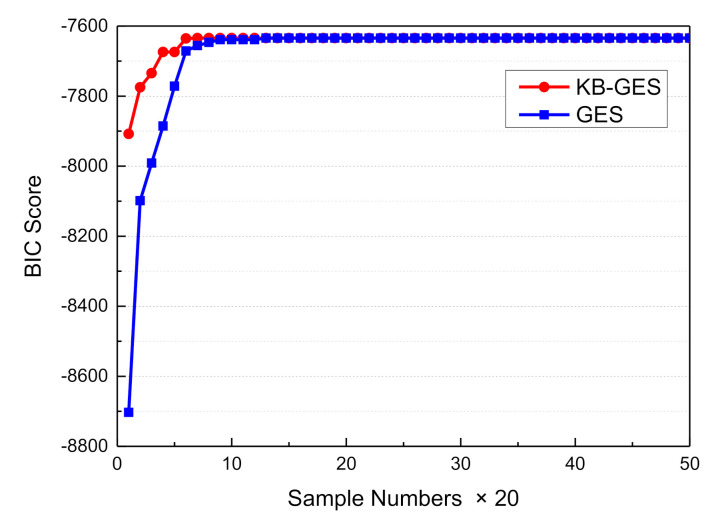
BIC score comparison.

Compared with the a priori network structure (Figure 19), the driving posture nodes (node11, node13, and node14) directly acted on the driving decisions mode node (node17). Meanwhile, the output of the decision node directly acted on the vehicle attitude, so that the attitude nodes (node18, node19, and node20) served as the diagnostic information. Due to the constraint of expert knowledge on the node directed arc, the meaningless edges that affected the decision variables node were removed, so that the search space dimension was effectively reduced and the search efficiency was improved. In this section, the modified BIC score (Equations (10) and (11)) was used to evaluate the obtained structure. Finally, we obtained a higher BIC score and the results are shown in Figure 23.

From Figure 23, the advantage of KB-GES under the condition of mined data learning is obvious, and the BIC score tends to be consistent with the increase of sample data. Therefore, we deemed the proposed KB-GES suitable for DBN structure learning, and obtained an accurate and reasonable structure that is closer to the priori Bayesian network.

Once the learned network structure was obtained, the next step was probabilistic reasoning. As shown in Algorithm 2, the main loop derives the scene eigenvalues and the vehicle attitude values, and then calculates the maximum posterior probability value of the decision mode node. Message propagation algorithms developed by Pearl are available in the literature [9]. Algorithm 2 shows the pseudo-code applied to implement the autonomous decision-making system on the driving simulator hardware platform.

**Algorithm 2** Pseudo-code of Bayesian probability programming**Input:** Observable Evidence Information**Output:** Decision Mode Confidence**Begin**: Preliminary Knowledge Initialization
**While (1)**
 Ground_truth = Discretize (CNN_OutPut && Sensor_read) Vehicle_attitude = Discretize (Sensor_read) Drive_mode (t) = Propagate (Ground_truth && Vehicle_attitude) Set_Maximum entropy principle (Drive_mode (t))
**End**


The purpose of DBN reasoning is to infer the probability of the maximum value of the query node. The confidence update rule of the decision node is:

(21) $$\mathrm{Bel}\left({\mathrm{Drive}}_{-}\mathrm{Mode}\right)=\alpha \lambda \left({\mathrm{Drive}}_{-}\mathrm{Mode}\right)\pi \left({\mathrm{Drive}}_{-}\mathrm{Mode}\right),$$

where α is a normalized factor applied to guarantee $\sum_{{\mathrm{Drive}}_{-}\mathrm{Mode}}\mathrm{Bel}\left({\mathrm{Drive}}_{-}\mathrm{Mode}\right)=1$. π means that the ground truth information is propagating forward along the directed arc, while λ means that the information is propagating backward along the arc. We assumed that the driver’s decision-making process is a stable random process in a finite space, and that the dynamic probabilistic propagation process is a Markov property that satisfies the following rule:

(22) $$P\left(X_{t+1}{|X}_{1},\cdot \cdot \cdot ,X_{t}\right)=\;P\left(X_{t+1}{|\;X}_{t}\right).$$

**Figure 24:**
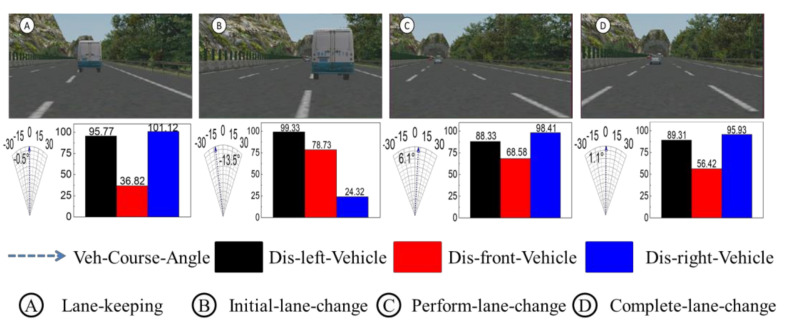
First left lane-change scene.

We took a vehicle performing the first lane-change as the test case. The synchronous traffic scene frames 140, 151, 163, and 170 are shown in Figure 24. As can be seen in the figure, the ego-vehicle drives in the first lane, and when the distance between the ego-vehicle and the front traffic vehicle reaches 36.8 m and an obstacle is present in the left lane at a distance of 95.8 m, the driver agent adjusts the vehicle attitude angle to perform left lane-change behavior. At frame 170, the ego-vehicle moves into the second lane and the full lane-change decision is then executed. This scenario demonstrates the variation of variables (course angle and longitudinal distance) at four frame slices.

**Figure 25:**
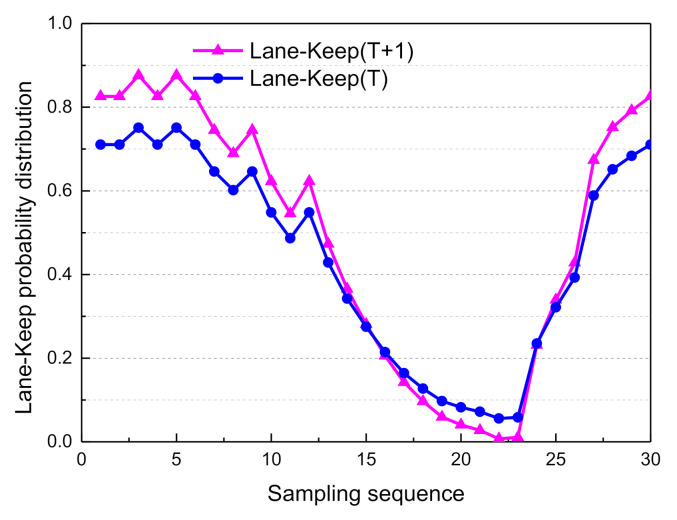
Adjacent moment probability distribution of lane keep.

**Figure 26:**
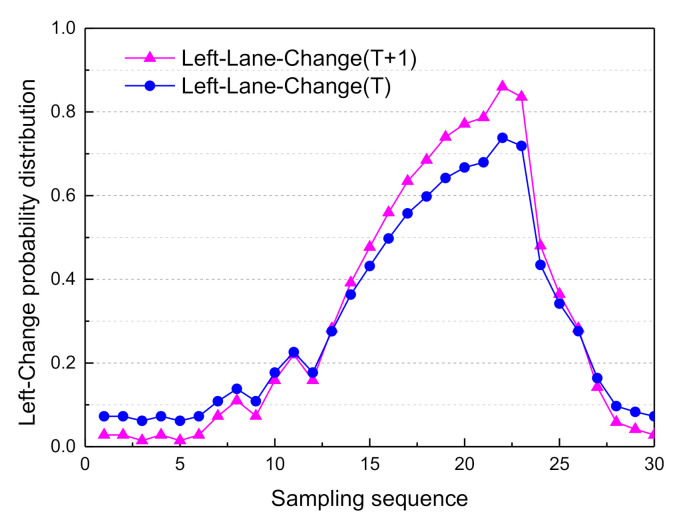
Adjacent moment probability distribution of left lane change.

**Figure 27:**
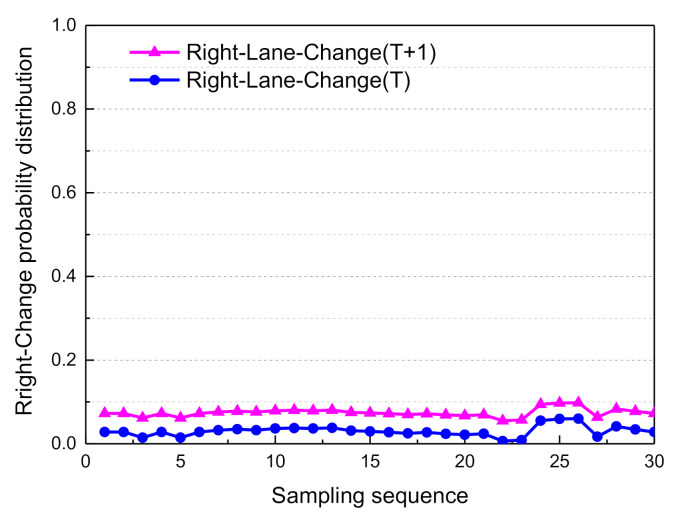
Adjacent moment probability distribution of right change.

**Figure 28:**
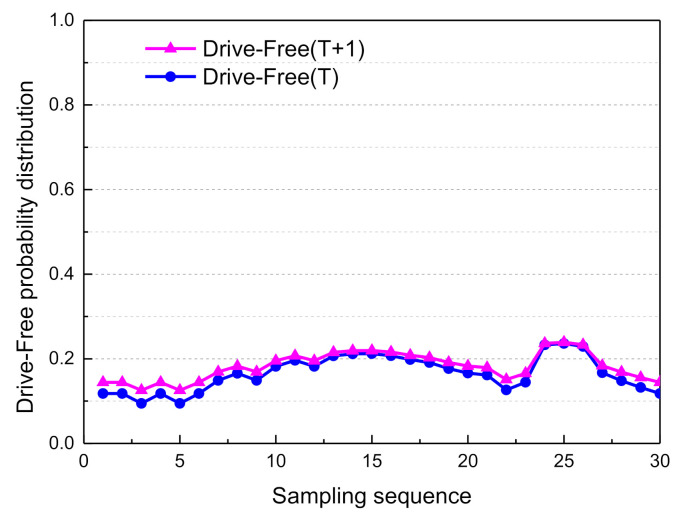
Adjacent moment probability distribution of free drive.

Based on the above Markovian assumptions in the probabilistic propagation algorithm, the decision mode confidence of adjacent moments could be obtained. The probability distribution curves of the driving decision modes are shown in Figure 25, Figure 26, Figure 27 and Figure 28.

From sampling points 1 to 10 of the T + 1th moment probability distribution curves of the driving modes, the probability distribution of Lane_Keep remains within 0.62–0.87; therefore, the lane maintenance mode is performed first.

From sampling points 11 to 23, when the front vehicle enters the safe area, the obstacle is in the left lane at a distance of 78.7 m (as shown in Figure 14), and the probability distribution of Left_Lane_Change gradually increases from 0.21 to 0.83. As a result, the left lane-change decision mode is executed. Subsequently, from sampling points 24 to 30, by adjusting the attitude of the vehicle so that it re-enters the lane-keeping mode, the probability distribution of Lane_Keep gradually increases from 0.23 to 0.82, and the entire lane change decision is then completed. Since the vehicle is in the first lane, and there is no right lane, it does not satisfy the right lane changing condition. Therefore, the probability distribution of right lane changing is less than 0.1. Considering the safety of vehicles and traffic regulations, the probability of free driving is less than 0.25.

**Figure 29:**
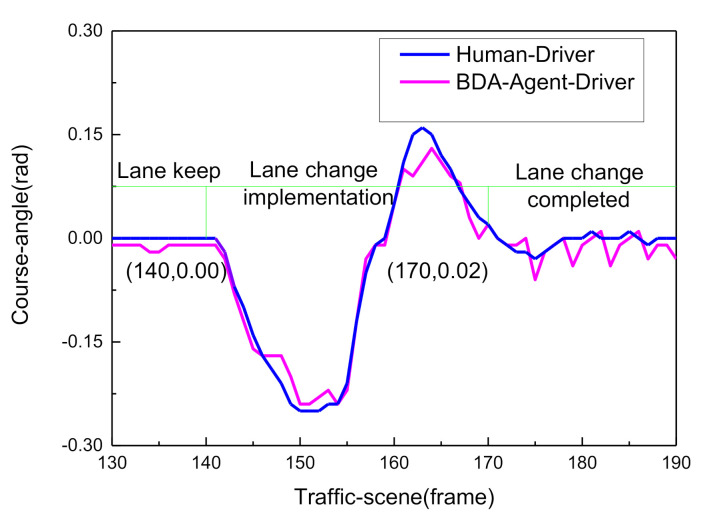
Comparative analysis of the BDA model decision and human driver’s intention.

In sum, the online real-time reasoning results are consistent with the offline simulation results (Figure 21), the experimental results verify the rationality and effectiveness of the DBN framework. The decision-making experience of human drivers is expressed by a probability distribution, and the model plenitude describes the driving behavior during the entire lane change process in typical urban road scenarios. As shown in Figure 29, since the vehicle course angle information embodies the driving decision intention, it can be used to evaluate the decision relevance. The similarity between the BDA model and the human driver’s decision intention was verified by calculating the intraclass correlation coefficient (ICC) [32,33]. 

As described in the reference [32,33], the author quoted the basic concept of ICC in the content of reliability analysis. So that in order to count the correlation between BDA model and Human-driver, we introduce ICC estimates and 95% confident intervals were calculated using SPSS statistical package version 23 based on single measures, absolute-agreement, one-way mixed-effects model, the results are shown in Table 6.

The intraclass correlation value 0.984 greater than 0.90 indicate excellent reliability according to the reference [32]. Based on the F-test one-way analysis of variance (ANOVA) with significance level α = 0.05, a total of 122 sample data of the two groups (n = 122, r = 2) were analyzed for variance. We can conclude that the p-value is greater than α = 0.05, which means there is a 95% certainty that the degree of variation between BDA model and Human-driver is significantly consistent.

## 4. Discussion

The goal of autonomous vehicle systems is to achieve brain-like decision-making. Our studies proposed a BDA model for autonomous vehicle system based on knowledge-aware and real-time data, which is used to learn the driver’s lane change decision process. As shown in Figure 14 and Figure 15 in Section 3.1, the longitudinal indicator MAE is less than 9 m and the horizontal indicator MAE is less than 0.5 m. In summary, the BDA agent model can accurately predict the indicator values of the complex urban road traffic scenes. This result implies that the model has a ability of cognitive understanding of traffic situations. It is also confirmed that the intraclass correlation coefficient between the BDA model and the human driver’s decision process reached 0.984, in other words, the BDA model can effectively predict the decision intention of human drivers, this enables autonomous agents to complete a series of basic driving tasks without human intervention. Although there are important discoveries revealed by this study, there are also some limitations.

First of all, the actual traffic scene is very complicated and it is hard to cover all cases in the simulation platform, this study only considers five urban roads with a total of 1000 traffic scenes, the generalization ability of the model needs to be verified in different road types and scene cases. Therefore, a large amount of effective data is an effective measure to improve the accuracy of prediction. As described in [34,35,36], driving models are learnt from large-scale video datasets.

Furthermore, our results show effectiveness of the driving policy model on the driving simulator platform, however, the model’s transformation from virtual to reality needs to be further optimized to adapt to realistic driving. As mentioned in [37,38,39], the article introduced a RL method for training neural network policy in virtual simulation and transferring it to a state-of-the-art physical vehicle system. Given realistic frames as input, driving policy trained by reinforcement learning can nicely adapt to real world driving situations.

## 5. Conclusions and Future Work

This study trained the convolutional network through the CNN multi-label task learning method, which provides an effective way to predict the indicator values of the complex traffic scene. It also introduced the Bayesian probability graph model. Based on qualitative and quantitative analyses of the driver’s decision-making process, we developed a KB-GES algorithm under the constraints of priori knowledge, which overcome the unexplainable characteristic of the end-to-end decision model. In summary, the proposed BDA model in this paper provides a new strategy to deal with human driver modeling.

It is also important that the constructed model should possess online self-learning and generalization capabilities. How to build an effective model to achieve these goals will be an interesting topic for future research. The following work will involve developing a model-based online reinforcement learning framework to optimize driving decision behavior based on safety cost functions and traffic rule constraint functions.

## Figures and Tables

**Table 1:** Structural parameters of the CNN layers.

Layer Name	Kernel Size	Stride	Tensor Size
Input Layer	Width × Height × Channels	-/-	231 × 231 × 3
Conv-1	11 × 11	4	56 × 56 × 96
Pool-1	3 × 3	2	27 × 27 × 96
Conv-2	5 × 5	1	27 × 27 × 256
Pool-2	3 × 3	2	13 × 13 × 256
Conv-3	3 × 3	1	13 × 13 × 384
Conv-4	3 × 3	1	13 × 13 × 384
Conv-5	3 × 3	1	13 × 13 × 256
Pool-5	3 × 3	2	6 × 6 × 256
FC-1	-/-	-/-	4096 × 1
FC-2	-/-	-/-	4096 × 1
FC-3	-/-	-/-	7
OutPut	S member function discretization status value		

**Table 2:** Traffic scene description factors and their interpretations.

Drive-Posture Description Parameter of Traffic Scenes
(1) Dis_front_Vehicle: Distance to the vehicle of the current lane
(2) Dis_right_Vehicle: Distance to the vehicle of the right lane
(3) Dis_left_Vehicle: Distance to the vehicle of the left lane
(4) Dis_left_Roadside: Distance to the left side of the road
(5) Dis_right_Roadside: Distance to the side of the road
(6) Dis_left_Lane: Distance between the left lane and left wheel
(7) Dis_right_Lane: Distance between the right lane and right wheel

**Table 3:** Driving decision semantic vectors and their interpretations.

Driving Decision Semantic Vector Space
(1) Veh_Speed: Vehicle longitudinal speed
(2) Veh_Acceleration: Vehicle longitudinal acceleration
(3) Veh_Course_Angle: angle between the axis of the vehicle body and road

**Table 4:** Driving mode variable and discretization values.

Query Node	State Description	Discretization Value
Driving decision mode	Left_Lane_Change	1
	Lane_Keep	2
	Right_Lane_Change	3
	Drive_Free	4

**Table 5:** DBN template model function layer and node description.

Layers of Model	Nodes of Model
Ground Truth	1. Dis_left_Vehicle, 2. Dis_front_Vehicle, 3. Dis_right_Vehicle, 4. Dis_left_rear_Vehicle, 5. Dis_rear_Vehicle, 6. Dis_right_rear_Vehicle, 7. Dis_left_Lane, 8. Dis_left_Roadside, 9. Dis_right_Lane, 10. Dis_right_Roadside
Situation Evaluation	11. ROI_front_situation, 12. ROI_rear_situation, 13. ROI_left_situation, 14. ROI_right_situation 15. Lane_Number, 16. Current_Lane
Driving Decision	17. Driving_decision_mode
Vehicle Attitude	18. Veh_Speed, 19. Veh_Course_Angle, 20. Veh_Acceleration

**Table 6:** Result of ICC Calculation in SPSS and F-test one-way ANOVA.

	Intraclass Correlation	95% Confidence Interval		F-Test One-Way ANOVA with α = 0.05				
		Lower Bound	Upper Bound	F-Statistic	Df1 (r-1)	Df2 (n-r)	*p*-Value	F-Critical One-Tail
Single measures	0.984	0.972	0.991	0.144	1	120	0.705	3.920
